# Comment on the exchange between Boddy *et al*. and Wagner *et al*.: malignancy, placentation and litter size

**DOI:** 10.1093/emph/eoaa027

**Published:** 2020-07-17

**Authors:** David Haig

**Affiliations:** Department of Organismic and Evolutionary Biology, Harvard University, 26 Oxford Street, Cambridge, MA 02138, USA

Data on comparative rates of cancer in different species are sparse. Boddy *et al*. analyzed 42 years of necropsy data from the San Diego Zoo for 37 mammalian species and found no relation between malignancy rates and lifespan, body mass or placental invasiveness, but found an association between malignancy rate and litter size. Wagner *et al*. question the conclusion that rates of malignancy are not related to placental type because Boddy *et al*. calculate the malignancy rate per necropsy whereas Wagner *et al*. would prefer the malignancy rate per tumor. Wagner *et al*. find a relationship between placentation type and malignancy rate using their preferred measure. In their response, Boddy *et al*. argue that these rates measure different things, and both are useful.

This has been a productive exchange. My own interest in comparative oncology has focused on the question whether mammals are particularly predisposed to cancer as a side-effect of maternal-fetal conflict over placentation and of evolutionary conflicts between genes of maternal and paternal origin within the placenta [1]. This hypothesis does not predict any simple relation between the degree of placental invasiveness and cancer rates for the reason identified by and colleagues: the depth of invasion is the outcome of opposing forces of placental intrusiveness and maternal defenses that can be resolved in different ways. For example, the porcine trophoblast is noninvasive in the uterus but invasive at ectopic locations [2]; the placentas of horses are epitheliochorial (non-invasive) but give rise to a detached cell population (chorionic girdle) that invades the endometrium and secretes hormones into the maternal circulation [3].

Boddy *et al.*’s finding that the malignancy rate per necropsy is positively related to litter size is of particular interest. Maternal–fetal conflict is expected to be more intense in species that produce litters than in species that produce singletons. The reason for this expectation is that the costs imposed by a segregating allele that increases fetal demand are borne by siblings without the allele, for competition within a litter, but are borne by future siblings, some of whom will inherit the allele, in species that produce singletons. Thus, the observation of a positive association of cancer rates with litter size is compatible with a conflict-based interpretation, although litter size correlates with other aspects of life-history which must also be considered.

The advantage of broad phylogenetic analyses is that these provide evolutionary replication for assessing correlations, but such analyses inevitably elide some of the fascinating biology of the individual species. The positive association of malignancy rate with litter size was partly driven by *Didelphis virginiana* (opossum) with a litter size of 9 and the highest cancer rate among the species in Boddy *et al.*’s study. Nine-banded armadillos (*Dasypus novemcinctus*), on the other hand, give birth to quadruplets but no malignancies were found in 67 total necropsies. Both these species raise questions on how litter size should be coded.

Boddy *et al*. used the number of young in a mother’s pouch as the litter size of opossums but one could argue that this underestimates litter size. Female opossums ovulate about 60 ova at once and pregnant females carry more than 20 embryos [4]. The reduction to the final litter size of fewer than 10 probably involves a neonatal scramble for a limited number of nipples augmented by selective removal of offspring from teats by mothers. Therefore, the intensity of sibling rivalry in opossum development is plausibly underestimated by the number of pouch young. The litters of nine-banded armadillos are monozygotic quadruplets [5]. In terms of the number of genetic individuals competing for maternal resources, this is a litter size of one.

The genetic-conflict interpretation would predict that cancer rates in nine-banded armadillos that produce monozygotic quadruplets should be similar to cancer rates in giant armadillos that produce monozygotic singletons, and both should be lower than euphractine armadillos that produce dizygotic twins (see [5]) whereas a standard life-history interpretation would relate cancer rates to the phenotypic litter size rather than the genotypic litter size. These are discriminating predictions, if only data were available on comparative cancer rates in armadillos!

**Conflict of interest:** None declared.
